# Recurrent Acute Otitis Media: What Are the Options for Treatment and Prevention?

**DOI:** 10.1007/s40136-017-0151-7

**Published:** 2017-05-09

**Authors:** Anna Granath

**Affiliations:** ENT-Department Karolinska University Hospital and Department of Clinical Science Intervention and Technology (CLINTEC), Karolinska Institutet, Stockholm, Sweden

**Keywords:** Recurrent acute otitis media, rAOM, Recurrent acute otitis media prevention, Recurrent acute otitis media treatment, Recurrent acute otitis media antibiotics, Recurrent acute otitis media vaccines, Adenoidectomy, Genetics

## Abstract

**Purpose of Review:**

To survey current strategies for treatment and prevention of recurrent acute otitis media (rAOM).

**Recent Findings:**

Treatment with systemic antibiotics is required in recurrent episodes of acute otitis media. A cautious attitude is recommended due to antibiotic resistance. Antibiotics also provide effective prophylaxis for rAOM. Topical treatment with ear drops is recommended in rAOM with otorrhea from tympanostomy tubes. Pneumococcal conjugated vaccines seem to have a moderate reductive effect on overall otitis media. The effect on rAOM is still unclear. Different administrations of immunoglobulins have not been effective against rAOM. Breastfeeding had a protective effect against rAOM. A recommendation against cigarette smoke exposure as a measure to prevent otitis seems warranted. An effect for adenoidectomy in children <2 years old with rAOM has been suggested. There is a strong genetic connection with rAOM. Probiotics and nasal spray with *Streptococci* might offer future opportunities as prophylaxis. Too little is known about complimentary treatments to give any recommendations.

**Summary:**

Systemic antibiotics are still needed as treatment against episodes of AOM in rAOM children. There are several preventive measures that can be taken to reduce the burden of AOM but they all have a small-moderate effect. Systemic antibiotics provide effective prophylaxis in rAOM, but must be used with extreme caution due to the emerging antibiotic resistance.

## Introduction

First described by Howie [1] as the otitis prone condition, we nowadays talk about recurrent acute otitis media (rAOM) as coined by Goycoolea [2]. The condition in a child is defined as having at least three episodes of acute otitis media (AOM) in a period of 6 months, or four or more episodes in 12 months. The microbiology is often more complex than in occasional episodes of AOM, but the most common bacteria are nevertheless *Streptococcus* *pneumoniae* (*Spn*), *Haemophilus influenzae* (*Hi*), and *Moraxella catharrhalis* (*Mcat*). This ailment will spontaneously resolve when the child gets older, often at 3–4 years of age. A child with rAOM brings a high strain on the family and prophylaxis against AOM episodes is highly requested. Surgery with tympanostomy tubes (TT), the most used prophylactic/treatment method, will be reviewed elsewhere in this issue.

## Treatment Options

When you discuss treatment for rAOM, it is important to first recognize that this condition warrants ambitious diagnostic procedures. Many children present with earache during an episode of upper respiratory infection (URTI), which almost always have a viral etiology. Redness of the tympanic membrane (TM) is a common finding which cannot be regarded to be exclusive for AOM. An accurate diagnosis might be difficult to achieve with otoscopy alone. You need to test the mobility of the TM with a pneumatic otoscope, or use tympanometry to establish if there is fluid in the middle ear. Infants have a floppy, narrow external ear canal, and inspection of the TM is often impossible without clearing the ear canal from earwax. Any physician, who has been challenged with the task of performing such a procedure in an agitated infant, knows that it can be very difficult. Children with suspected or established rAOM are often referred to an ear, nose, and throat (ENT) department/practice. The first measure is to verify the diagnosis. This is especially important in order to be able to refrain from unnecessary antimicrobial treatment, or surgical procedures with TT. The family also requests advice on how to manage the situation with a child with frequent painful AOM episodes, and they usually have questions about the outcome regarding hearing loss and side effects of antibiotic intake. It is highly recommended not to involve too many doctors, in order to maintain continuity, because such an attitude will in all probability provide a higher diagnostic accuracy. It may also be a useful course of action to offer the family emergency (daytime) appointments when the child has symptoms of AOM, in order to establish what kind of ear ailment the child is suffering. Systemic antibiotics are frequently used as treatment for sporadic episodes of AOM as well as rAOM. The emerging problem with antibiotic resistance among bacteria warrants a cautious attitude towards subscription of antibiotics. There is also a recently largely recognized impact of viral infections in AOM, and a strong connection with episodes of URTI [3, 4, 5••]. This might explain the relatively modest effect of systemic antibiotics against overall AOM [6], since much of the symptoms emanate from the viral URTI and not from the AOM episode. There is a lack of evidence regarding drug efficacy for antibiotic treatment specifically in rAOM children. One study investigated the effect of levofloxacin [7] in children with a high risk of AOM or history of rAOM. The protocol was open label without placebo, or comparing standard AOM antibiotics (for instance amoxicillin). Levofloxacin is a fluoroquinolone, a group of antibiotics that are much associated with promoting antibiotic resistance in bacteria. These drugs have also been deemed unsuitable in young children, due to a possible risk for musculoskeletal side effects. Another similar open-label study [8] evaluated the effect of cefdinir, an extended spectrum cephalosporin in children with high risk for AOM. It is safe to say that broad spectrum cephalosporins are highly questioned nowadays because of their supposed connection with development of extended spectrum beta-lactamases in bacteria. Recent randomized controlled studies (RCTs) with a stringent diagnostic protocol show that there is a larger benefit with antibiotic treatment (amoxicillin with clavulanic acid) in more severe cases of AOM [9, 10] in children <2 years old. Significantly, more adverse events in children treated with antibiotics were also noted. These studies are more relevant for rAOM children than many older investigations, due to the homogenous age group. Children with rAOM were one specific randomization group in the first study [9]. In the second [10], the range of reported previous episodes of AOM was up to ten episodes at 23 months of age, indicating rAOM. None of the studies did however report any subgroup analysis on individuals with rAOM. In clinical practice, rAOM often results in several cures of systemic antibiotics. Antimicrobial therapy will for the nearest future continue to be the treatment of choice in rAOM children for symptomatic episodes of AOM. It is also highly likely that rAOM children with mild episodes of AOM might be looked upon as any child with an occasional episode of AOM, and a watchful waiting can be applied. The microbiological background is more complicated in rAOM and will be reviewed elsewhere in this issue. A more active approach towards microbiological diagnostics in cases of rAOM is recommended in order to avoid treatment failure and to detect development of antibiotic resistance. Such diagnostics include microbiological cultures from ear discharge and/or from the nasopharynx. Penicillin and amoxicillin (with or without clavulanic acid) will suffice in most cases. A recent study on 5- vs 10-day treatment with amoxicillin with clavulanic acid [11] favored the 10-day course. The study did not include any specific report on subgroups of children with rAOM, and there is a lack of clinical evidence regarding the duration of intervention with antibiotics in OM.

Children with TTs due to rAOM often have otorrhea as a sign of AOM episodes. There is evidence [12, 13] that topical treatment with combined glucocorticoid-antibiotic ear drops most often will suffice as treatment in these cases, and that the use of systemic antibiotics is unnecessary.

## Prevention

### Socioeconomic Factors, Breastfeeding and Parental Smoking

There are many reports on the influence of socioeconomic factors on the occurrence of OM in various populations with challenging living conditions [14–18]. These studies present results on the whole panorama of OM. Less is known about the specific implications of poor-living conditions, deficient health services, and low levels of parental education on young children with rAOM. One research group studied an indigenous Filipino population with a high risk of OM [19•]. When comparing genetic factors to environmental ditto, they found that the genetic disposition with a specific genotype was a stronger risk factor than the socioeconomic status. An epidemiological survey from Denmark [20] has recently presented data on risk factors for early otitis media, before 6 months of age, which is recognized as predicting factor for rAOM [21, 22].

The Danish study [20] included 69,105 mothers and their children. The final analysis of data revealed that the following factors were predictive of early otitis media: male gender, prematurity, parity (number of pregnancies the mother has had), maternal age, maternal self-estimated health, maternal intake of penicillin during pregnancy, and termination of breastfeeding before the child was ≥6 months old. One possible information bias in this study was that the study subjects were requested to understand and speak Danish at a certain level. This might have left out immigrant mothers with skewing of data as a result. The importance of breastfeeding for the development of rAOM has been much discussed, with the assumption that the immune factors provided within the breast milk (secretory IgA, antimicrobial proteins, fatty acids, and cytokines) [23] have a protective effect against URTI and AOM. There are data supporting that breastfeeding for at least the first 4 months of life can reduce the risk of early AOM [4, 18, 24–26], but in the Filipino cohort above [19•], there was no association between breastfeeding and OM, as in an early study on rAOM [22]. Parental smoking has also been listed as a risk factor for various types of respiratory problems and URTIs, including AOM. A Norwegian survey [27] showed that maternal smoking during pregnancy increased the risk (RR 1.34) for early OM, and also a slightly increased risk for rAOM (RR 1.24). Some previous studies [18, 25, 28–30] have also showed a slightly increased risk for AOM with smoke exposure, although there also have been results suggesting that parental smoking is insignificant [22]. Day care attendance is another suggested risk factor, which is attributed to frequent infections, of mostly viral origin, among young children and their families [31]. Exposure to high number of other young children naturally leads to an increased frequency of URTIs that can cause episodes of AOM. Epidemiological studies confirm that day care attendance, and as a consequence introduction to a high-risk environment regarding URTI, is associated with AOM [18, 25, 28, 30]. Colonization with potential AOM-pathogen was also increased in children with early onset OM, who had siblings in day care [32]. An earlier study [22] did however not find that day care attendance was a risk factor for developing rAOM, but that the condition had already manifested itself before the children went to kindergarten.

### Genetics

It is empirical knowledge among many physicians who meet children with rAOM that the ailment often runs in the family. It also seems from a clinical point of view that the family history can be predictive about the outcome for the child, regarding further development of rAOM. There is also scientific evidence that there is a strong genetic component for OM [33–35]. Experimental mice models with deficient Toll-like receptor 4 have previously been identified as especially susceptible to OM [36, 37]. In a clinical setting, this is now also shown for certain populations [38••]. There are also other genetic components for OM that are being revealed at a fast pace, both in experimental models [39] and in humans [40•, 41, 42]. One research group found differences in gene regulations of innate immune responses between children with rAOM and controls [43••]. Interestingly, the same group also recently has discovered deficiencies in mucosal antibody responses in rAOM [44]. The knowledge about the heritability of OM is useful when meeting patients. Even if it does not provide us with a solution to the problem, it may provide the patients and their families with, an explanation. There are to date no suggested genetic tests or advisory guidelines regarding genetics in OM.

### Antibiotics

The rAOM condition implies that the affected individuals suffer from repeated middle ear infections of bacterial origin. It therefor seems obvious that antibiotics could be used as prophylaxis against rAOM. Lately, it has become evident that viral upper respiratory tract infections are a prerequisite for the development of AOM [3]. In the light of this paradigmatic shift, the efficacy of antibiotics as treatment and prophylaxis must be scrutinized. Early studies on young children with rAOM showed that antibiotics could provide protection against OM recurrence and frequency of AOM episodes [45–47], with amoxicillin appearing as the drug of choice. In a recent review comparing studies on antibiotic prophylaxis, adenoidectomy, and tympanostomy tube insertion (TT) [48], antibiotic prophylaxis proved to be the most effective preventive treatment. There are to date no suggestions on antiviral drugs that could be used as prophylaxis for OM.

### Vaccines

In a recent Cochrane review, the effect of pneumococcal conjugate vaccines (PCV) as prevention for otitis media [49••] was evaluated. Nine studies were included, of which five studies surveyed intervention at an early age (<1 year) in healthy infants, and four studies included older children (ages 1–7 years) who were either healthy or had a history of respiratory disease, with or without OM. In total, 48,426 children participated in the vaccine trials. The vaccine types were either 7-valent PCV (with polysaccharides from serotypes 4, 6B, 9V, 14, 18C, 19F, and 23F) with CRM197 carrier protein (CRM197-PCV7, six trials), 9-valent PCV (serotypes 1, 4, 5, 6B, 9V, 14, 18C, 19F, 23F) conjugated to CRM197 (CRM197-PCV9, one study), 7-valent with carrier protein OMPC (OMPC-PCV7, one study), or 11-valent (serotypes 1, 3, 4, 5, 6B, 7F, 9V, 14, 18C, 19F, 23F) conjugated a surface lipoprotein from *Hi*, protein D (PD-PCV11, one study). In addition, one study on CRM197-PCV7 also included a booster vaccination with a 23-valent pneumococcal polysaccharide after infancy. Another study compared the effect of trivalent influenza vaccine (TIV) with or without PCV7 to hepatitis B vaccine and placebo. The primary outcome measure was frequency of all-cause otitis media. The CRM197-PCV7 had a modest effect with a relative risk reduction of 7% (RRR) when administrated during the first year of life to children with a low risk of OM. The reduction was larger for CRM197-PCV7, OMP-PCV7, and PD-PCV11 when OM specifically caused by *Spn* (*Spn-OM*) was studied (RRR 20–52%). For PD-PCV11, there was an additional reduction in OM caused by NTHI. The trials on older children showed a reduction of all-cause OM for CRM197-PCV9. The administration of CRM197-PCV7 to older children (with a history of URTI and AOM) did not lead to a reduced risk of AOM, nor did the combination with a booster of 23-valent pneumococcal polysaccharide. The study including TIV showed that influenza vaccine alone might have a larger preventive effect on AOM than when combined with CRM197-PCV7. There was a decrease in *Spn*-OM among the older children, but it was not statistically significant. The effect on rAOM was also evaluated. There was an effect (RRR 9–10%) for CRM197-PCV7 for the younger age group. The decrease for rAOM in the PD-PCV11 study was not significant. The four trials on older children did not report on rAOM. The results in the review were not pooled to perform a meta-analysis, due to clinical diversities among the included trials. One RCT on infants with a [50] high risk of rAOM was performed. The results were favorable towards vaccination with a 26% reduction of AOM episodes in the vaccine group. The study was not included in the Cochrane review [49••] due to methodological reasons (not blinded).

Pneumococcal conjugated vaccines with protein D from *Hi* as carrier protein have caught special attention regarding rAOM, since there is a high representation of *Hi* as a causative agent. The effect of such a vaccine, the 10-valent PHiD-CV, which is a successor to the vaccine designated PD-PCV11 above, has been evaluated in the last years. A large clinical trial was performed in several Latin American countries [51•] including approximately 24,000 children with main aims to establish the vaccine effect against community-acquired pneumonia and clinically confirmed otitis media. Out of the main cohort, 7359 children (Panama only) were randomized to participate in the otitis media trial. In the end, 3010 (PHiD-CV) and 2979 (controls) were evaluated. The protocol for OM diagnosis was ambitious and based on clinical diagnosis by skilled physicians. One setback was that there were fewer cases of OM than anticipated, which reduced the power of the study. The vaccine effect regarding OM was 16% for clinically confirmed cases, and 67% for vaccine serotype-specific OM. The outcome for OM caused by *Hi* was not significant. A Finnish RCT including children <1 year old was performed to evaluate the effect of PHiD-CV on the frequency of tympanostomy tube placements (TTP) [52•]. The research group found a vaccine effect of 13% regarding reduction in TTPs (number needed to vaccinate 44 to avoid one TTP), which however failed to reach significance. The same group analyzed the effect of PHiD-CV on outpatient purchases of antimicrobial drugs [53•] and found a vaccine effect of 7–8% regarding any antimicrobial drug and antimicrobial drugs for OM, respectively. The number needed to vaccinate to avoid one antimicrobial drug purchase was five. To date, a 13-valent pneumococcal conjugated vaccine has been introduced, CRM197-PCV13 (serotypes 1, 3, 4, 5, 6A, 6B, 7F, 9V, 14, 18C, 19A, 19F, 23F), and replaced CRM197-PCV. A study on the sequential introduction of PCV (PCV7➔PCV13) in Israel [54] showed a reduction of OM, both *Spn*-OM with vaccine type *Spn* (VT) and non-*Spn*-OM in children <3 years old. The authors speculate that the reduction in non-*Spn*-OM might be due to PCV protecting against early OM which could potentially result in more complex OM. A study from Australia [55] focused on OM and a recent shift between PHiD-CV and CRM197-PCV13. Introduction of PHiD-CV was previously reported to result in reduced frequency of suppurative OM among children from socioeconomically challenged communities [56, 57]. Disappointingly, introduction of the broader vaccine did not further improve ear health. The vaccine efficacy for prevention of invasive disease has been proven [58, 59]. Although thoroughly studied regarding several aspects, including OM, there is at present a lack of knowledge on the specific effects of PCV on rAOM.

### Adenoidectomy

Adenoidectomy is since long in clinical use as adjuvant surgery in OM, with or without tube insertion. An effect of adenoidectomy on OME has been proven [60]. There has been diverging results concerning the effect of adenoidectomy on rAOM. In one study, adenoidectomy was evaluated on young children (*n* = 180) with rAOM [61]. The participants were randomized to either of three treatment arms: adenoidectomy, antibiotic prophylaxis (sulfafurazole), or a placebo drug. The follow-up time was 24 months and failure was defined as two episodes of AOM in 2 months or three episodes in 6 months, or middle ear effusion persistent for >2 months. At 24 months, treatment failure was similar in all three groups. Similar results evolved from another study [62]. A recent systematic review and meta-analysis [63•] compared TT insertion alone with combined surgery with TT and adenoidectomy. Fifteen studies were included, main outcome measure was repeated surgery with TT and r-TT. The meta-analysis revealed a protective effect of combined surgery against r-TT in children older than 4 years with rAOM or otitis media with effusion (OME). The protective effect was not seen in children less than 4 years of age. Another systematic review [64••] investigated adenoidectomy with or without grommets with non-surgical treatment or grommets only. Primary outcome was failure at 12 months after surgery, defined as four or more episodes of AOM, presence of effusion for >6 months, need for additional surgery, or hearing improved <10 dB. There were fewer failures at 12 months in the adenoidectomy group. Further analysis showed that two groups specifically had a favorable effect of adenoidectomy: children <2 years old with rAOM (16% failure rate vs 27% with or without adenoidectomy, respectively) and children ≥4 years with persistent OM.

### Experimental Studies

There have been many candidate treatments for the prevention of rAOM. Xylitol, a natural sugar substitute, can reduce the adherence of *Spn* and *Hi* to nasopharyngeal cells in vitro and has been tested in several trials. A review on this subject [65] concluded that there might be a moderate effect on overall AOM, but there was not enough evidence on the effect during URTI or in rAOM. Another theoretical approach is the application of bacteria, alpha-hemolytic *Streptococci* (AHS), which could have an inhibitory effect on *Spn* and *Hi*. A RCT using treatment with a nasal spray containing AHS [66] did not reveal any significant effect on the frequency of AOM. Another RCT on a spray containing *Streptococcus salivarius* 24SMB was more successful and showed a certain effect in reducing episodes of rAOM in children that were colonized with these bacteria [67]. Probiotics have also been a candidate lately. One study, a RCT comparing orally consumed probiotics to placebo did not prove an effect regarding reducing AOM episodes in rAOM children [68]. *Lactobacillus rhamnosus* GG was found in the adenoid of children who underwent adenoidectomy after an oral course of probiotics [69•], and in another study in middle ear fluid [70]. The clinical implications of these findings are still unclear. Biofilm formation in the middle ear in rAOM and other types of longstanding OM has been identified as a contributing factor. An experimental substance, dornase alpha, was recently presented as a potential future treatment candidate against biofilm formation [71].

### Immunoglobulins

Substitution with immunoglobulin was tested with systemic administration in a controlled trial on rAOM children [72]. There was no reduction of AOM or URTI in the treated group. Another study tested topical treatment with intranasal application of IgG [73] on children <2 years old with TT due to rAOM. The treatment failed to reduce the number of otorrhea episodes, and did not affect nasopharyngeal colonization with *Hi*. One can interpret the favorable outcome of early breastfeeding as a natural type of immunomodulation with supply of secretory IgA.

### Complimentary Medicine

Complimentary treatments tend to vary depending on local traditions and how strong the support for these alternative treatments is in the local medical society. There are reports on several treatments, for instance chiropractic, osteopathy, and herbal medicine. One review on this matter [74] concluded that there is no sufficient evidence on these treatments, and that further studies are warranted before any recommendations can be given. There are examples of interesting experimental studies, for instance on the effect of oil of basil and essential oils on experimental otitis media [75]. One recent open-label randomized Japanese trial tested the effect of juzen-taiho-to (JTT), a traditional Japanese herbal medicine, on children with rAOM [76]. The JTT group had a significantly lower number of AOM episodes and coryza, plus a reduced rate of antibiotic administration. One weakness of this study was the open-label protocol.

## Discussion

Regarding treatment for episodes of AOM in rAOM children, systemic antibiotics remain an important option, even though watchful waiting might be applied in mild cases. Studies on treatment in AOM supply, some support for the use of antibiotics in rAOM even though they do not exclusively investigate children with rAOM. For tube otorrhea, topical treatment with combined glucocorticoid and antibiotic ear drops is recommended as a first choice, and is now also shown to be better than observation only [13]. The mechanism for the efficacy of topical treatment in tube otorrhea is not fully known. A protective effect of topical ear drops against external otitis media and possibly biofilm formation on the tubes is one theory. It is not known if surgery with TTs in rAOM children results in a lower use of antibiotics for AOM. There is a need for further studies on this matter, but the recommendation for topical treatment in cases if tube otorrhea might at least facilitate a shift for treatment with less systemic antibiotics. As for the topic of prevention of rAOM, extensive research on vaccines against *Spn* and *Hi* has been performed over the years. We still do not have a vaccine against *Hi* that provides highly effective protection against AOM. The existing vaccines are directed against the capsulated *Hi* bacteria, and it has been difficult to develop an exclusive vaccine against non-encapsulated *Hi*. Regarding *Spn* the development of PCV has been successful, especially against invasive infections like meningitis and septicemia. The effect on AOM has also been extensively evaluated. PCV can reduce overall AOM to a certain degree, and has a better effect against AOM caused by vaccine-specific serotypes of *Spn*. The addition of *Hi*-specific protein-D seems to lower AOM caused by *Hi*. The effect on AOM was only seen when the vaccine was introduced at an early age. There are also some indications that introduction of PCV has led to a lower frequency of surgery with TTs, and a lower purchase of systemic antibiotics. The effect of PCV on rAOM-populations is still unclear, although it is possible that PCVs can have a slight reductive effect. Recent findings imply that rAOM children have an immunologically poorer answer to PCV than average [77].

Adenoidectomy has a proven effect on OME in older children, and is often recommended as adjuvant surgery in re-TT procedures. Recent reviews present somewhat contradicting results. New data however suggests that adenoidectomy might have a protective effect against AOM on rAOM in children ≤2 years of age. As a conclusion, adenoidectomy can be considered as an adjuvant surgical procedure in rAOM children with clinically significant signs and symptoms of a large adenoid, for example obstructive nighttime breathing problems.

Regarding socioeconomic factors, the evidence for recommending parents to take special measures is strongest for breastfeeding for at least 4 months. To avoid exposure to cigarette smoke also seems to have some impact on the occurrence of rAOM. These two advices are also widely accepted as child health promoters in a wider perspective. Day care attendance also affects the frequency of AOM episodes, but the rAOM condition most often develops before the child goes to kindergarten. It is also a very large decision to remove a child from day care, and it is probably an example of when the measure will not quite meet the means.

In a larger perspective socioeconomically challenged populations would benefit from better living conditions and provision of good healthcare. This is related to not only AOM but also a wider child health perspective, and the effect specifically on rAOM is uncertain due to the strong genetic connection. It is not within the power of the practicing physician to change such circumstances. Neither is it meaningful to advice parents to change their circumstances, since we must assume that parents want what is best for their children, and will do what they can to provide a healthy environment.

Preventive treatments with probiotics look promising in clinical studies, and can hopefully evolve to effective clinically applied prevention methods. There are also some progresses regarding topical intranasal treatment with safe bacteria that can inhibit AOM pathogens.

Regarding complimentary treatments, we still need more knowledge before considering any guidelines.

Knowledge of the genetic component might in a near future help us to give more personalized advice, and perhaps perform genetic tests and supply treatments that are better suited for each individual together with better individual surveillance regarding complications.
